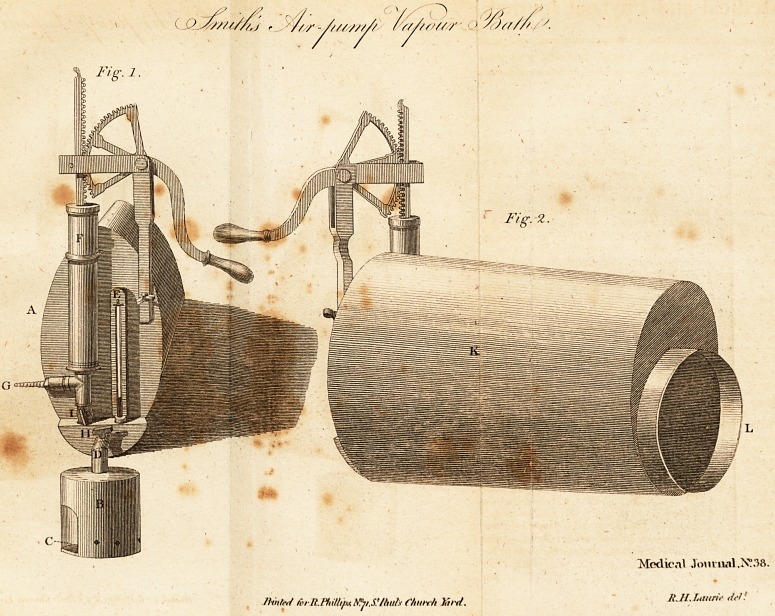# Mr. Blegborough, on the Vapour Bath

**Published:** 1802-04

**Authors:** Ralph Blegborough

**Affiliations:** Member of the Royal College of Surgeons. Oxford Street


### Medical and Physical Journal.

VOL. VII.] APRIL, 1802. [NO. XXXVIII.

### TO DR. BRADLEY.

DEAR SIR,

I Send you, for your valuable Publication, the plate of a machine for conveying a vapour bath to diseased limbs, and for taking off the pressure of the atmosphere; which, if I am not deceived in its effects, is calculated to prove of the greatest use in changing the action of diseased parts, little doubting but it will rank among the first of the modern improvements in medicine, and, under the superintendance of professional men, prove a powerful means not only of alleviating, but frequently curing, many diseases which have been considered incurable.

As the machine comprehends, and, for the first time, combines the application of fomentation and the cupping-glass, two of the most powerful external means of acting on disease, and that on a plan more extensive than was ever before thought of; what effects may we not be led to expect from so extended a combination? Every day's experience is proving to us the efficacy of the application in gout, rheumatism, contractions of muscles, cutaneous and other diseases, particularly of the chronic kind; and though its application has not yet been so extensive, under my own immediate inspection, as to warrant my affirming it to the extent I hope to be soon able to do; nevertheless I have reason to believe, from some recent cases, that acute diseases also are not beyond the reach of its influence, and that from its power in promoting a copious diaphoresis and relaxation of the whole system, it may be made to produce the happiest effects in phrenitis, pleuritis, enteritis, &c.

The peculiar construction of the machine before us is adapted to the leg or arm only, but the principle extends much further, and can be applied to any part of the body by the intervention of glasses, or otherwise. One is already made that includes one half of the body; and when we consider that by its means the pressure of the atmosphere can be removed from so large a surface, every square inch of which sustains a weight of 15 lb. the effects on the vessels circulating the fluids in such parts are too evident to need insisting on in this Work. One very very obvious one is, that the consequent temporary expansion of them from so great a pressure being removed, must give an opportunity for obstructions formed in them to give way, at the same time arresting the progress of inflammation, the means which Nature, left to herself, is under the necessity of employing to bring about the same end. In this manner is avoided a great deal of misery arising from symptomatic fever, &c. and not unfrequently destruction of parts, the consequence of suppuration.

Though this is the first drawing that has been made of it, or the first time any thing has appeared concerning it in print, yet the credit of it is by no means to establish. It is too well founded in philosophy and good theory not to have attracted the attention of medical men, some of whom, high in their profession, have not only recommended it to their patients, but have had it applied to their own persons with the most marked benefit. Many valuable cases are already in my possession, some of them of personages of the first consideration in this kingdom; but as it is my intention to publish an account of them, I shall content myself at present with simply stating its principles, and leave it with your numerous readers to draw their own conclusions.

EXPLANATION of the PLATE.

FIG. 1. A view of that end of the machine to which the exhauster, &c. are affixed. (A) the body of the machine. (B) the boiler containing the fluid, the fumes of which are thrown into the machine through the stop-cock (H), and heated by means of (C) a spirit lamp. (D) the valve in the boiler for the escape of the fumes when prevented entering into the machine by turning the stop-cock (H). (E) a thermometer, shewing the temperature of the fumigation. (F) the exhauster for exhausting the machine after the fumigation has continued a proper time, 40 strokes of which are as many as any person can bear at once. (G) the escape valve of the exhauster; to which a flexible pipe is adapted to convey the air from the chamber, if vitiated by the nature of the affection for which the application is made. (H) the stop-cock. (I) another, to prevent the valves of the exhauster from being injured by the hot fumes as they enter the machine during the fumigation.

FIG. 2. A perspective view of the machine from the other end, where the limb is admitted. (K) The body of the machine made of strong copper tinned in the inside, tin itself not being equal in all cases to the pressure of the atmosphere. (L) The mouth of the machine, to which is attached a strong bladder, cut so that one end of it may go over, and be firmly secured to it by means of a ligature, the other so that it may be drawn over the thigh, when passed through it into the machine, and secured upon it by means of a roller.

Mr.

Mr. Smith, of Brighton, a Member of the Society of Arts, whofe mechanical genius is well known to the world, claims the merit of its invention, and has, by occafional alterations in the fpace of fome years, brought it to its prefent ftate of fimplicity and perfection. The idea of fucking poifon from wounds firft led him to believe that mechanical means might be employed on the fame principle to a great extent. How far the intention is anfwered by the air pump vapour bath, and how extenfive the application of it may be made in the treatment of difeafe, the public are to be the judges.

I fhall add nothing more at prefent than a letter to the Proprietor from Dr. Hamilton, Phyfician to the London Difpenfary, who accidentally faw the effects produced by its application.

"Dear Sir,

"I have often, fince the time I examined your machine for conveying a vapour bath to difeafed limbs, and for taking off the preffure of the atmofphere, confidered it and its probable effects with much attention; and my opinion is, that it is likely to be of the greateft ufe to mankind, in helping obftructed veffels to unload themfelves. The mode in which it muft act is agreeable to found theory, and the more it is put in practice the oftener will facts occur to confirm what I now fay. I not only view it as valuable to remove local affections of the extremities, but alfo to afford relief in other parts of the body, where obftructions may have taken place. There are fome complaints which would often be greatly mitigated, if not always removed, by its power in producing a general and copious fweat over the whole frame, fuch as in the diabetes, dropfy, and other maladies where the fkin is parched, dry, and hard. In general, it will be well to pump out the air gradually, and to fee the effects of a partial exhauftion of the machine; for its being done too fuddenly, would allow the air in the circulating fluids to exert its fpring with too great force, producing acute pain, &c. The machine, by having a condenfing pump fixed to it, might be ufed as a bracer, and that with beneficial and powerful effects. Was I not fo far advanced in life, and fo much engaged in other avocations, I would with gladnefs have devoted my time to the ufe of it, thoroughly convinced that it would prove ufeful to the diftreffed, and honourable to myfelf. Wifhing you, dear Sir, the favour of God, which is better than life and all that life contains, I am

*No. 7, Artillery Place,* Your friend, and obedient fervant,
*April* 8, 1801. JAMES HAMILTON."

Profeſſional Gentlemen and others, who have made Philoſophy an object in their reſearches, will have an opportunity of inſpecting the machine itſelf, at No. 330, Oxford Street, and at Mr. James's, No. 5, Cumberland Place, New Road, Mary-le-Bone. I am, &c.

RALPH BLEGBOROUGH,
Member of the Royal College of Surgeons.

*Oxford Street, Feb. 10, 1802.*

## Figures and Tables

**Fig. 1. Fig. 2. f1:**